# The Effectiveness of Myo-Inositol and D-Chiro Inositol Treatment in Type 2 Diabetes

**DOI:** 10.1155/2016/9132052

**Published:** 2016-10-11

**Authors:** Basilio Pintaudi, Giacoma Di Vieste, Matteo Bonomo

**Affiliations:** ^1^Diabetes Unit, Niguarda Cà Granda Hospital, 20162 Milan, Italy; ^2^Diabetes Unit, Cantù Hospital, 20081 Abbiategrasso, Italy

## Abstract

Inositol has been used as a supplement in treating several pathologies such as PCOS, metabolic syndrome, and gestational diabetes. Both myo-inositol and its isomer d-chiro-inositol showed insulin mimetic effects in conditions of insulin resistance. Type 2 diabetes (T2DM) is a condition typically caused by insulin resistance. There is a lack of evidence of inositol use in T2DM. We evaluated the effectiveness and safety of myo-inositol and d-chiro-inositol treatment in T2DM. This was a pilot study involving a consecutive sample of patients with T2DM with suboptimal glycemic control (HbA1c 7.0–10.0%) already treated with glucose-lowering agents. Patients (23.1% males, mean age of 60.8 ± 11.7 years) took for three months a combination of myo-inositol (550 mg) and d-chiro-inositol (13.8 mg) orally twice a day as add-on supplement to their glucose-lowering drugs. Possible occurrence of side effects was investigated. After three months of treatment fasting blood glucose (192.6 ± 60.2 versus 160.9 ± 36.4; *p* = 0.02) and HbA1c levels (8.6 ± 0.9 versus 7.7 ± 0.9; *p* = 0.02) significantly decreased compared to baseline. There was no significant difference in blood pressure, lipid profile, and BMI levels. None of the participants reported side effects. In conclusion, a supplementation with a combination of myo- and d-chiro-inositol is an effective and safe strategy for improving glycemic control in T2DM.

## 1. Introduction

Inositol is a cyclitol present in animal and plant cells. It can be present in nine distinct stereoisomers, myo-inositol being the most represented. D-chiro-inositol is an inositol isoform derived from myo-inositol through an epimerization process and this reaction is insulin dependent [1]. Both myo- and d-chiro-inositol showed insulin mimetic effects in animal models of insulin resistance [2, 3]. They have been studied in basic research and their potential has been recognized as very promising [4]. A specific physiological myo-/d-chiro-inositol ratio exists in different human tissues [5]. The distinctive ratio depends on the specific biological function these molecules have. Indeed, looking at the molecular pathway of inositols, after insulin links with the cell, inositol-second messengers are produced. While d-chiro-inositol-based second messengers promote glycogen synthesis, second messengers based on myo-inositol regulate glucose intake increasing the activity of glucose transport proteins. Recently, a new supplement formulation conforming to this physiological ratio has been designed [6, 7]. Given inositol pivotal role in regulating many metabolic pathways and hormonal signalling, its use in clinical settings is steeply growing. Inositol has been mainly used as a supplement in treating several pathologies such as PCOS [8], metabolic syndrome [9, 10], and gestational diabetes (GDM) [11]. In the case of GDM, a condition defined as a glucose impairment first detected in pregnancy, a preventive role of inositol for GDM onset was recognized [12–14]. In addition, inositol has been studied as a therapeutic option for the treatment of GDM [15, 16]. The main effect of inositol when used in pregnancy is decreasing the level of insulin resistance. Consequently, a potential role of inositol as a treatment option could be hypothesized for other conditions typically characterized by insulin resistance. The most common disease in which insulin resistance represents a significant factor is type 2 diabetes mellitus (T2DM). It is characterized by a double pathophysiological alteration represented on one side by an increase in insulin resistance, that is, the inability of the action of blood circulating insulin, due to a resistance of the target tissues; on the other side, by a deficiency of insulin secretion from the pancreas [17]. The fist-line therapy for treating T2DM is metformin, an insulin sensitizer drug [18, 19]. However, the natural history of the disease implies that the therapy with only one oral hypoglycemic agent may be no longer able to guarantee adequate glycemic compensation. It therefore becomes necessary to start a therapy with a combination of other glucose-lowering drugs and, in the case of failure of oral therapy, to start insulin [20, 21]. There is a lack of evidence of inositol use in T2DM. Inositol could represent a valid strategy in treating T2DM in addiction to hypoglycemic drugs. Aim of our study was to evaluate the effectiveness and safety of myo-inositol and d-chiro-inositol treatment in T2DM.

## 2. Materials and Methods

This was a pilot study involving people with T2DM cared for at the diabetes outpatient unit of the Niguarda Cà Granda Hospital, Milan, Italy. A consecutive sample of patients with T2DM treated with at least one glucose-lowering agent was included in the study, irrespective of gender and diabetes duration. Patients were not referred to the center at the time they entered the study; they instead had received a medical continuous assistance, being treated at the same center for at least one year. Subjects were included in the study if their glucose status was steadily suboptimal for at least 3 months. Suboptimal glycemic control was defined as a glycated hemoglobin (HbA1c) between 7.0% (53 mmol/mol) and 10.0% (86 mmol/mol). Other inclusion criteria were age ≥ 18 years, unchanged glucose-lowering treatment in the last 3 months, and informed consent signature. Exclusion criteria were the presence of renal or liver diseases, severe heart failure, psychiatric disease, and pregnancy.

To all the involved subjects a combination of myo-inositol (550 mg), d-chiro-inositol (13.8 mg), and folic acid (400 mcg) (INOFOLIC® Combi, soft gel, Lo.Li. Pharma, Rome, Italy) was suggested to be taken orally twice a day as add-on supplement to their glucose-lowering drugs. All participants, as standard procedure at each visit, were properly advised to follow a hypocaloric and low-glycemic index diet according to standard care and to maintain their standard physical activity. For all patients a three-month follow-up visit was scheduled. Clinical information at baseline and after three months visits was recorded. Specifically, information on the following parameters was collected: age, gender, diabetes duration, height, weight, smoke habits, blood pressure levels, HbA1c levels, lipid profile, pharmacologic treatments (antihypertensive, lipid-lowering, and glucose-lowering drugs), and diabetes-related complications such as retinopathy, nephropathy, neuropathy, and macroangiopathy. In particular, macroangiopathy was defined as a history of a cardiovascular event and/or ischemic electrocardiogram abnormalities at rest or in a stress test, or the presence of plaques detected by ultrasonographic examination of the carotid arteries or the peripheral arterial vessels or as the presence of an intima media of thickness >1.5 * *mm [22]; neuropathy was diagnosed by the vibration perception test, the monofilament pressure sensation test, or electromyography; nephropathy was defined as an increased urinary albumin excretion (albuminuria) diagnosed if urinary albumin concentration was >30 * *mg/l, or if urinary albumin excretion rate was >20** *μ*g/min, or if urinary albumin-to-creatinine ratio was >2.5* * mg/mmol in men and 3.5* * mg/mmol in women; and retinopathy was detected by high-quality fundus photographs [23].

Possible occurrence of side effects related to the consumption of inositol was investigated at each visit. All the clinical data collected in the study have been analyzed anonymously. Local Ethic Committee approved the protocol and all participating subjects gave a written informed consent. The study was conducted according to the Helsinki Declaration.

### 2.1. Statistical Analyses

Data were expressed as means ± standard deviation for continuous variables and percentages for categorical variables. The Kolmogorov–Smirnov test was used to test the normality of distribution of continuous variables. Clinical and demographic characteristics were compared using the Wilcoxon signed-rank test. As this was a feasibility study, no prior power calculation was performed. A* p* value < 0.05 was considered for statistical significance. Analyses were performed using SPSS version 21.0 (SPSS, Inc., Chicago, IL).

## 3. Results and Discussion

Overall, 20 subjects with T2DM were involved in the study. The acceptance rate of participation at the study of patients to which it was proposed was 100%. Demographic and clinical characteristics of the studied patients are reported in Table 1. One-fourth of participants were male. Almost half of the entire population had diabetes-related complications. Comorbidities such as antihypertensive and lipid disorders were present in almost half of the cases. With respect to diabetes treatment 53.8% of subjects were treated with only oral hypoglycemic agents (OHA), 38.5% with OHA plus insulin, and 7.7% with only insulin. After three months of treatment fasting blood glucose levels (192.6 ± 60.2 versus 160.9 ± 36.4, *p* = 0.02) and HbA1c levels (8.6 ± 0.9 versus 7.7 ± 0.9, *p* = 0.02) significantly decreased compared to baseline. HbA1c reduction was of −1.0 ± 1.5%. There was no statistically significant difference in systolic (*p* = 0.10) and diastolic (*p* = 0.90) blood pressure, lipid profile (*p* = 0.31 for total cholesterol; *p* = 0.89 for HDL cholesterol; *p* = 0.61 for triglycerides; *p* = 0.69 for LDL cholesterol), and BMI levels (*p* = 0.14) changes (Table 2). None of the participants reported side effects linked to the consumption of inositol.

The main finding of our study is that inositol could represent a valid strategy for improving glycemic control in T2DM, its supplementation being effective in lowering both fasting blood glucose and HbA1c levels. We cannot compare our results with existing literature because of the lack of studies involving people with T2DM treated with inositol. There is only evidence that both myo-inositol to chiro-inositol epimerase activities and chiro-inositol to myo-inositol ratios are decreased in tissues of GK type 2 diabetic rats, potentially playing a role in explaining the decreased chiro-inositol to myo-inositol urine and tissue ratios observed in animal and human studies [24]. This mechanism may also possibly play a role in explaining insulin resistance status. A tissue-specific myo-inositol/d-chiro-inositol ratio exists. This characteristic is strictly linked to the different biological actions that inositol isoforms exert, myo-inositol being more effective in increasing the insulin sensitivity level of typically insulin-dependent tissues (e.g., muscle, fat) and d-chiro-inositol more involved in tissues where glycogen synthesis takes place (e.g., liver). We did not use myo-inositol or d-chiro-inositol alone because we decided to administer inositol complying with a proportion that should reflect the natural balance among the two stereoisomers. This new formulation reflects the myo-inositol/d-chiro-inositol ratio of 40 : 1 that is the physiological ratio we can find in the plasma.

Our study was conducted involving people with T2DM, one of the largest global health emergencies with a huge economic impact. Its prevalence is increasing worldwide and it is expected that it will increase up to 7.7% in 2030. Each year more and more people live with this condition, which can result in life-changing complications. In addition to the 415 million adults who are estimated to currently have diabetes, there are 318 million adults with impaired glucose tolerance, which puts them at high risk of developing the disease in the future [25]. People with diabetes are at high risk of developing a number of disabling and life-threatening health problems. Diabetes complications can be prevented or delayed by maintaining blood glucose, blood pressure, and cholesterol levels as close to normal as possible. Several studies have shown a clear association between strict glucose control and a low risk of diabetes-related complications [26, 27]. The greatest clinical objective on both the part of health care professionals and patients is maintaining HbA1c in target, thus limiting the occurrence of complications.

The result of HbA1c reduction in our study was quite surprising because of the short period of treatment and because of the extent of the reduction. OHA can improve HbA1c levels with a mean HBA1c reduction of 1% [28, 29]. We found that a very important inositol effect on glycemic control exists. The reason for this could be likely linked to a lowering of the insulin resistance status thanks to the metabolic effect of inositol. However, we cannot exclude the possibility that a part of this effect could be associated only with lifestyle changes such as more proper diet and physical activity. We can exclude a putative effect on blood glucose control of folic acid contained in the supplement, as a systematic review and meta-analysis confirm [30].

Although our study was a pilot study, despite the small sample size, it reached a statistical significance in the two main parameters (i.e., blood glucose and HbA1c) defining glycemic control. Another novelty of our study was having tested the metabolic effect of inositol supplementation in a population including also males. The studies until now published on inositol have involved only women with several clinical (i.e., PCOS, metabolic syndrome, and GDM) or physiological (i.e., menopause) conditions. Only a single randomized, double-blind, placebo-controlled study involved a sample of males with T2MD but it was focused on investigating myoinositol use in the treatment of erectile dysfunction [31]. However, authors did not specifically investigate parameters linked to glycemic control. Our findings could extend the target population for inositol use also to males, its supplementation being effective in improving metabolic parameters. As an effective insulin sensitizer inositol could represent a possible alternative to metformin or pioglitazone that are typically used as insulin sensitizer glucose-lowering drugs, when their use is not possible (i.e., metformin intolerance, drugs contraindications).

Having carried out the study in the context of an usual care setting, we were not able to have information on insulin-resistance levels, serum insulin being a parameter not usually requested and used. This represents a limitation of our study.

## 4. Conclusions

Our study is important for several reasons. It showed for the first time a direct beneficial effect of the supplementation with the association of myo- and d-chiro-inositol on glycemic parameters of subjects with T2DM. Particularly, a significant reduction in blood glucose and HbA1c levels was registered. Inositol supplementation did not lead to any side effect, confirming the safety of this molecule. Our study findings are relevant, mostly for their possible clinical implication. Our data are promising but they need to be confirmed by studies with a larger sample size and a randomized controlled trial design.

## Figures and Tables

**Table 1:** Baseline demographic and clinical characteristics of the studied patients.

Gender (%)	
Male	23.1
Female	76.9
Age (years)	60.8 ± 11.7
Diabetes duration (years)	11.5 ± 7.6
Smokers (%)	23.1
Diabetes complications (%)	
None	53.8
Retinopathy	15.4
Nephropathy	15.4
Macroangiopathy	15.4
Neuropathy	0.0
Feet	0.0
Antihypertensive treatment (%)	53.8
Lipid-lowering treatment (%)	53.8

**Table 2:** Clinical changes between baseline and three months after starting inositol treatment.

	Baseline	3 months	*p*
Weight (kg)	80.5 ± 17.7	79.8 ± 15.8	0.14
BMI (Kg/m^2^)	31.1 ± 5.9	30.9 ± 6.6	0.14
Classes of BMI (%):			0.34
<25 Kg/m^2^	15.4	20.0	
25–29 Kg/m^2^	23.1	10.0	
≥30 Kg/m^2^	61.5	70.0	
Systolic blood pressure (mmHg)	126.7 ± 10.8	124.4 ± 9.5	0.10
Diastolic blood pressure (mmHg)	75.0 ± 7.7	76.1 ± 5.5	0.90
Fasting blood glucose levels (mg/dl)	192.6 ± 60.2	160.9 ± 36.4	0.02
HbA1c (%)	8.6 ± 0.9	7.7 ± 0.9	0.02
Total cholesterol (mg/dl)	189.3 ± 39.5	198.7 ± 38.8	0.31
HDL cholesterol (mg/dl)	57.3 ± 19.6	50.6 ± 10.1	0.89
Triglycerides (mg/dl)	161.5 ± 131.1	149.9 ± 64.4	0.61
LDL cholesterol (mg/dl)	98.9 ± 36.1	116.4 ± 43.7	0.69
